# Bio-Oss^®^/Avitene™ composite scaffold promotes maxillofacial bone regeneration via early osteoimmunomodulation of BM-MSCs: an *in vitro* and clinical study

**DOI:** 10.3389/fbioe.2026.1795343

**Published:** 2026-06-10

**Authors:** Maria Rosa Iaquinta, Raffaella De Pace, Antonio D’Agostino, Fabio Lonardi, Lorenzo Trevisiol, Guido Lobbia, Assia Benkhalqui, Alessia Finotti, Giulia Breveglieri, Mauro Tognon, Fernanda Martini, Elisa Mazzoni

**Affiliations:** 1 Department of Medical Sciences, University of Ferrara, Ferrara, Italy; 2 Department of Translational Medicine, University of Ferrara, Ferrara, Italy; 3 Unit of Oral and Maxillofacial Surgery, Department of Surgical Sciences, Dentistry, Gynaecology and Paediatrics, University of Verona, Verona, Italy; 4 Centre for Medical Sciences (CISMed), University of Trento, Trento, Italy; 5 Department of Life Sciences and Biotechnology, Section of Biochemistry and Molecular Biology, University of Ferrara, Ferrara, Italy; 6 Laboratory for Technologies of Advanced Therapies (LTTA), University of Ferrara, Ferrara, Italy

**Keywords:** Bio-Oss^®^/Avitene^™^, bone marrow derived-mesenchymal stem cells (BM-MSCs), bone regeneration, inflammatory response, maxillofacial surgery

## Abstract

Inflammation is an important biological process to be considered in designing successful biomaterial-based therapeutics. Indeed, prolonged inflammation can result in delayed wound healing and, in some cases, may cause the rejection of the biomaterial together with additional tissue damage. Mesenchymal stem cells (MSCs) participate in this critical intercellular communication to modulate bone healing. In this study, MSCs were employed as *in vitro* model in order to evaluate the biological performances, in terms of immune response, of Bio-Oss®/Avitene™ composite scaffold. Moreover, new bone formation was evaluated in patients undergoing maxillomandibular osteotomy for skeletal malocclusion, facial asymmetry, or aesthetic indications, using a Bio-Oss®/Avitene™ composite scaffold. The early inflammatory response of human bone marrow derived-mesenchymal stem cells (BM-MSCs) was investigated *in vitro* analysing the gene expression of IL-6 and IL-8 using Droplet Digital PCR and the release of cytokines, chemokines, and growth factors using Bio-Plex approach. Clinical evaluation was performed using Cone Beam Computed Tomography (CBCT). *In vitro*, Bio-Oss®/Avitene™ decreases the IL-6 expression, quantified with Droplet Digital PCR approach. Bio-Plex reveals that the protein levels of MCP-1 and IL-6 were reduced, while IL-4, IL-8 and VEGF were increased by the scaffold in cells up to day 7. CBCT assessment in patients demonstrated stable outcomes, supporting the potential for long-term aesthetic restoration of the zygomatic region. These findings indicate that Bio-Oss®/Avitene™ possesses immunomodulatory potential and is capable of directing anti-inflammatory, immune-mediated responses associated with bone regrowth for the treatment of dentofacial deformities through orthognathic surgery.

## 1 Introduction

Functional and aesthetic facial rehabilitation represents, at present, a key focus in maxillofacial surgery, as balanced facial features and natural proportions play a crucial role in supporting self-esteem and psychological health. Maxillomandibular osteotomy is a well-established surgical approach for correcting skeletal malocclusions, realigning the sagittal and vertical axes of the upper incisors, and improving the aesthetic contour of the lower third of the face, including the paranasal region, lips, and chin. In this context, biomaterials, or scaffolds, play a predominant role becoming a viable treatment for bone abnormalities. Given that the tissue microenvironment is crucial in steering cellular fate towards either homeostasis, repair, or disease, biomaterials have been developed specifically to mimic the structure, composition, and function of the tissue microenvironment to repair or replace damaged or diseased tissues and organs (De Pace et al., 2025). Previous studies investigated hydroxyapatite–collagen composite scaffolds both *in vitro*, where they induced osteogenic differentiation of adipose-derived mesenchymal stem cells (hASCs) (Mazzoni et al., 2017; Iaquinta et al., 2022) and in patients who underwent zygomatic augmentation with prostheses in combination with maxillomandibular osteotomy (D’Agostino et al., 2016; Mazzoni et al., 2020). The other study confirmed these findings in a clinical setting in a large cohort of patients undergoing malar augmentation, reporting high volumetric stability, good integration with the underlying bone and histological evidence of new bone formation (D’Agostino et al., 2016). Clinical studies have demonstrated the safety and biocompatibility of synthetic bone graft materials. In this context, the hydroxyapatite-collagen composite (HA/Col) has shown excellent tolerance in patients, with no serious complications or significant inflammatory responses observed. Reported adverse effects were minimal, mild, and easily manageable, confirming a favorable safety profile. These findings indicate that HA/Col not only effectively promotes bone regeneration but does so without triggering local inflammatory reactions (Li et al., 2019). Overall, these results indicate that the hydroxyapatite–collagen composite is a safe and effective material for bone regeneration and reconstructive applications.

Inflammation is also an important biological process to be considered in designing successful biomaterial-based therapeutics. Studies that investigate the interplay between the biomaterial and the host’s inflammation process are crucial because modulation of the immune microenvironment has emerged as a key factor shaping the success of bone regeneration (Li et al., 2025). After implantation, biomaterials interact with the host immune system and trigger a cascade of biological events collectively known as the foreign body response (FBR). This response begins with the recruitment of immune cells such as neutrophils, macrophages, and dendritic cells. These early interactions strongly influence the subsequent cellular response and determine whether the implanted material will integrate with the surrounding tissue or lead to adverse reactions (Oluwole et al., 2024). Acute inflammation represents the initial physiological reaction following implantation and is characterized by the rapid infiltration of neutrophils and macrophages, which remove debris and initiate tissue repair. However, if the inflammatory stimulus persists, the process may evolve into chronic or prolonged inflammation, leading to fibrous capsule formation, and potential implant failure (Hortensius and Harley, 2016; Corduff and Goldie, 2025). The ability of a biomaterial to regulate this transition is therefore crucial for successful tissue integration and regeneration.

Several *in vitro* studies have demonstrated that the physicochemical properties of biomaterials including surface chemistry, topography, and porosity, can modulate macrophage polarization and the immune microenvironment. For instance, modifications in biomaterial nanostructure have been shown to influence macrophage behavior, promoting polarization toward an anti-inflammatory M2 phenotype and enhancing regenerative responses (Zheng et al., 2022). Nano-hydroxyapatite biomaterials have been reported to regulate macrophage polarization and reduce inflammatory cytokine production while stimulating osteogenic factor expression, highlighting the concept of osteoimmunomodulation in bone tissue engineering (Xu et al., 2019). Mesenchymal stem cells (MSCs) participate in critical inter-cellular communication or cross-talk to modulate bone healing. MSCs actively secrete cytokines, chemokines, and growth factors that act either on themselves by an autocrine loop or neighbouring cells, the paracrine loop, to modulate the immune system, and inflammatory response, as well as stimulating neo-angiogenesis. Bone progenitor MSCs not only differentiate into bone, but also interact with the immune system to promote the healing process. The close relationship between the immune and skeletal systems has been referred to as “osteoimmunology” (Iaquinta et al., 2022). In addition to differentiating into bone, bone progenitor MSCs work with the immune system to aid in the healing process. The efficacy of MSCs in bone regeneration depends on a complex bidirectional “crosstalk” with immune cells, in which MSCs use their immunomodulatory properties to transform a hostile inflammatory location into a favorable regenerative environment. MSCs act as central coordinators that manage the inflammatory microenvironment, which is crucial because bone regeneration is an immune-driven process. Mesenchymal stem/stromal cells modulate the immune response through interactions with innate immune cells. Through the secretion of factors like PGE2, IL-10, and TGF-β, MSCs promote the polarization of macrophages from the M1 phenotype (pro-inflammatory/destructive) to the M2 phenotype (anti-inflammatory/reparative). MSCs modulate B-cell maturation and antibody production, further stabilizing the local environment and promote the development of T-regulatory (Treg) cells, which shield the newly formed tissue, while inhibiting the growth of pro-inflammatory Th1 and Th17 cells. In addition, the MSCs function as a “Bio-pharmacy” release a sophisticated “secretome” that includes: chemokines and cytokines to attract and guide immune cells (ii) growth factors: (e.g., VEGF, IGF-1, BMPs) to promote bone mineral deposition and blood vessel creation, angiogenesis. Exosomes are tiny vesicles that transport proteins and microRNA to nearby or distant cells contributing to tissue regeneration activity (Oluwole et al., 2024).

In our previous study, Bio-Oss®/Avitene™ scaffold was assessed employing an *in vitro* cellular model of primary human adipose stem cells (hASCs), in order to assess its biological proprieties (Iaquinta et al., 2022). In particular, Bio-Oss®/Avitene™ scaffold can influence the hASC immune response during cell osteogenic differentiation, by modulating cytokine and chemokine expression (mRNA), leading to a decrease in the pro-inflammatory IL-6 and promoting an immunomodulatory response favourable to osteogenesis *in vitro*. These findings support the concept that biomaterials can regulate not only cell differentiation but also immune cell behaviour (Iaquinta et al., 2022).

The first study showed that this scaffold can induce osteogenic differentiation of hASCs, promoting the expression of osteogenic markers and supporting new bone formation with good biocompatibility (Iaquinta et al., 2022). Thus, the aim of this study is to comprehensively investigate the modulation of the BM-MSCs immune response *in vitro* by analysing the secretome, in order to elucidate the mechanisms underlying the bone regeneration observed in maxillofacial patients.

## 2 Materials and methods

### 2.1 Scaffold composition and fabrication

The innovative material Bio-Oss®/Avitene™ was tested *in vitro* and in patients. The composite scaffold, used herein, is composed of 1 mm–2 mm bovine spongious, bone substitute Bio-Oss® granules (Geistlich Biomaterials, Thiene, Italy) mixed with Avitene™ Microfibrillar Collagen Hemostat (Bard Warwick, Rhode Island) (Iaquinta et al., 2022). Specifically, the Bio-Oss® granules (3 g) were combined with collagen Avitene™ (1 g), followed by 6 mL of sterile water. The mixture from the combination of Bio-Oss® granules and collagen Avitene™ was used to obtain small disks (Ø, 1 cm; height, 0.2 cm) for the *in vitro* analysis: disks were left to dry overnight under UV light (Iaquinta et al., 2022). The procedure to obtain the handmade prostheses to clinical implantation is describe in paragraph 2.6.

### 2.2 Scanning electron microscopy analysis

The structure of Bio-Oss®/Avitene™ composite scaffold was analysed by scanning electron microscopy (SEM). To this purpose, BM-MSCs were grown on scaffold up to day 7. After cells culture, scaffolds were washed with PBS 1x and fixed for 1 h by 2.5% glutaraldehyde and 4 h by 1% osmium solution in phosphate buffer. Samples were sputter-coated with gold and SEM analysed (Cambridge United Kingdom, model Stereoscan S-360) (Mazzoni et al., 2017).

### 2.3 Cell culture and seeding onto scaffolds

In this study*, in vitro* analyses were performed using human BM-MSCs. BM-MSCs at the second passage, were bought as cryopreserved frozen cells from Lonza Milan, Italy (PT-2501). BM-MSCs are guaranteed to differentiate down the chondrogenic, osteogenic and adipogenic, lineages when cultured in specific conditions. BM-MSCs expressed CD44, CD105, CD166 CD29 and did not express CD14, CD34, and CD45 markers. Cells were grown in αMEM (Lonza, Milan, Italy) supplemented with 10% fetal bovine serum (FBS) (Lonza, Milan, Italy), antibiotics, and incubated at 37 °C in a humidified environment with 5% CO_2._ Primary BM-MSCs were maintained in αMEM basal medium and grown (i) on the Bio-Oss®/Avitene™ biomaterial and (ii) in tissue culture polystyrene (TCPS) vessels (24-well plates) used as control at a density of 5 × 10^3^ cells/well (Mazzoni et al., 2020). In Osteogenic Conditions (OC), BM-MSCs were exposed to differentiation Bullekit osteogenic medium (Lonza, Milan, Italy), composed by osteogenic basal medium (Lonza, Milan, Italy) and osteogenic SigleQuotes, which includes dexamethasone, ascorbate, mesenchymal cell growth supplement, L-glutamine, and -glycerophosphate (Lonza, Milan, Italy) (Iaquinta et al., 2021). Scaffold fractions were separately arranged in 24-well plates (Ø, 10 mm) to cover the surface area, filled with 200 µL of cell suspension containing 2 × 10^4^ BM-MSCs for each sample and incubated for 2 h. Then, a volume of basal medium up to 1 mL was added. Cells were re-fed with fresh medium every 3 days until the time of analysis.

### 2.4 Droplet digital PCR

RNA was isolated using the RNeasy Plus Micro Kit (Qiagen, Milan, Italy), according to the manufacturer’s instructions, at day 7. Total extracted RNA was quantified by using a Nanodrop spectrophotometer (ND-1000; Wilmington, Delaware). Purified RNA from BM-MSCs that were grown on Bio-Oss®/Avitene™ scaffold and on TCPS was reverse transcribed to cDNA using the ImProm-II Reverse Transcriptase (Promega, Milan, Italy). Total cDNAs were stored at − 20 °C until the time of droplet digital PCR (ddPCR) analyses. To allow for more specific, analytical detection of Interleukin (IL)-6 and IL-8, the QX200 ddPCR System (Bio-Rad Laboratories, Segrate, Italy) was used for ddPCR analyses (Torreggiani et al., 2019).

Briefly, ddPCR was performed in a total volume of 22 μL by adding 11 μL of 2X ddPCR Supermix for Probes (No dUTP) (Bio-Rad, Milan, Italy, Cat. N. 1863024), 1 μL of the specific IL-6 and IL-8 PCR Assay (Hs00174131_m1_FAM and Hs00174103_m1_FAM, respectively; Cat. N. 4331182, ThermoFisher, Milan, Italy), 10 ng of cDNA, and nuclease-free water. After droplet generation inside the Automated Droplet Generator (Bio-Rad Laboratories, Segrate, Italy), the 96 well-plate was heat-sealed with foil and placed in the SimpliAmp Thermal Cycler (Thermo Fisher Scientific, Milan, Italy). PCR conditions were: 95 °C for 10 min, then 40 cycles at 94 °C for 30 s and 55 °C for 1 min (ramping rate of 2 °C/s), and two final steps at 98 °C for 10 min and a 4 °C indefinite hold to enhance dye stabilization. The analysis was performed in triplicate for each experimental group.

### 2.5 Bio-Plex detection proteins

Cytokines, chemokines and growth factors in culture supernatants released from cells under analysis, were measured by Bio-Plex Pro Human Cytokine 27-plex Assay (Catalog number M50-0KCAF0Y, Bio-Rad Laboratories, Segrate, Italy) (Fabbri et al., 2015), as described by the manufacturer, at day 3 and 7. Samples were analysed on a Bio-Rad 96-well plate reader using the Bio-Plex Suspension Array System and Bio-Plex Manager software (Bio-Rad Laboratories, Segrate, Italy). Data were analysed using the Bio-Plex Manager Software (Bio-Rad Laboratories, Segrate, Italy), and cytokine concentrations were reported as pg/ml.

### 2.6 Clinical study design and patient evaluation

The Ethics Committee of our University Hospital approved the clinical study, protocol number 3731CESC. The study included n = 30 out of n = 65 consecutively treated patients who underwent combined ortho-surgical treatment for different types of dento-facial deformity. They all met the inclusion and exclusion criteria. Inclusion criteria were the following: (1) patients diagnosed with different type of malocclusions, with no distinction in gender or age; (2) patients who needed malar augmentation as part of surgical treatment, (3) patients who underwent bimaxillary orthognathic surgery. Exclusion criteria were the following: (1) patients with previous history of facial trauma; (2) patients who had already undergone bimaxillary surgery; (3) patients diagnosed with bone illnesses involving both the facial and the body skeleton; (4) patients who presented incomplete medical record at the time of data collection (for example, lack of CTs scans).

Patients underwent orthognathic surgery at the Unit of Maxillofacial Surgery of Verona University Hospital between 2020 and 2022. Diagnosis, surgical planning, surgery, and follow-up assessment were all performed by the same expert ortho-surgical team (Arnett et al., 2022a; 2022b; Trevisiol et al., 2023). Planning of surgical movements was based on the Face-Airway-Bite (FAB) principles as described by Arnett et al., FAB protocol used for profile and frontal treatment (Arnett et al., 2022a; 2022b). The handmade prostheses were obtained by mixing 3 g of Bio-Oss® (1 mm–2 mm) with 1 g of collagen Avitene™ followed by 6 mL of sterile water (Iaquinta et al., 2022) Shaping was performed according to surgical needs, depending on the clinical evaluation of the patient. The mixture obtained from the combination of Bio-Oss® granules and collagen Avitene™ was used to obtain two almond-shaped zygomatic prosthesis (15 mm × 5 mm x 7 mm). Biomaterials were blended at the beginning of the surgical session, and let dry under light for 3 hours, then inserted at the end of the surgery. After osteosynthesis, before the closure of the surgical access, using the same upper vestibular incision of Lefort I with a subperiosteal dissection of the area between the infraorbital nerve medially and the zygomatic arch laterally, a pocket was created over the zygomatic bone, which was similar-sized to the prosthesis, the latter was then positioned without any need of fixation (Iaquinta et al., 2022). The mean dimension of the prostheses was calculated on patients’ Cone Beam Computed Tomography (CBCT) (NewTom VGI EVO, Qr Verona, Cefla). For 3D reconstruction of the inserted prosthesis a free software 3D Slicer was used (Fedorov et al., 2012).

### 2.7 Statistical analysis

Data were presented as mean ± standard deviation (SD). Statistical analyses of biological experiments (n = 3), which were performed in technical triplicate, were carried out by using GraphPad Prism 8.0 software (GraphPad Software, La Jolla, CA, USA). A p-value <0.05 was considered significant (*); p-value <0.001(**), p-value <0.0001 (***) was considered highly significant. The statistical differences in the expression of IL-6 and IL-8 (mRNA) and released cytokines were determined by one-way ANOVA analysis for repeated measures and Tukey’s multiple comparison test. Scientific and Research plot tool (SRplot) (Tang et al., 2023) for data visualization was also used.

## 3 Results

### 3.1 Scanning electron microscopy of Bio-Oss®/Avitene™ biomaterial

**FIGURE 1:**
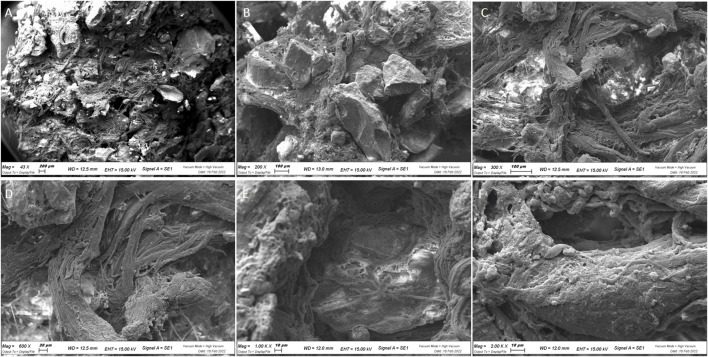
Scanning electron microscopy analysis of Bio-Oss®/Avitene™. Bovine collagen fibrils from Avitene™. Microfibrillar Collagen Haemostat were mixed with bovine Bio-Oss to generate the final scaffold. The structure of the scaffold was SEM analyzed at different magnification. Scale bar: 200 μm, × 43 **(a)**, Scale bar: 100 μm, ×200 **(b)**, Scale bar: 100 μm, ×300 **(c)**, Scale bar: 20 μm, ×600 **(d)**, Scale bar: 10 μm, ×1.00k **(e)**, Scale bar: 10 μm, ×2.00k **(f)**. SEM lower magnifications (×43 and ×100) remark the structure of the hydroxylapatite more clearly than higher magnifications **(a,b)**. A fibrous structure appears when collagen fibers and granular HA are combined, together with collagen fibers of composite scaffold, at 300 x-2.00k magnifications **(c-f)**. Cells were spread on the substrate confirming that the composite scaffold offers a good microenvironment for BM-MSC adhesion and proliferation **(f)**.

SEM analysis was performed to investigate the microstructure of the composite scaffold. Bovine collagen fibrils from Avitene™ Microfibrillar Collagen Hemostat were mixed with bovine Bio-Oss to generate a highly fibrous structure (Figure 1). The composite scaffold exhibited collagen fibers’ microstructural features, such as fusing/bifurcating fibrils, rotates at a porous structure of hydroxylapatite. In addition, some splaying, such as twisted fibers around one another and mixed branching of fibers, was seen (Figures 1c,d). Finally, SEM analysis showed that cells were spread on the substrate confirming that the composite scaffold offers a good microenvironment for BM-MSC adhesion and proliferation (Figure 1f).

### 3.2 IL-6 and IL-8 pro-inflammatory cytokines expression (mRNA) in BM-MSCs grown on Bio-Oss®/Avitene™ biomaterial

**FIGURE 2:**
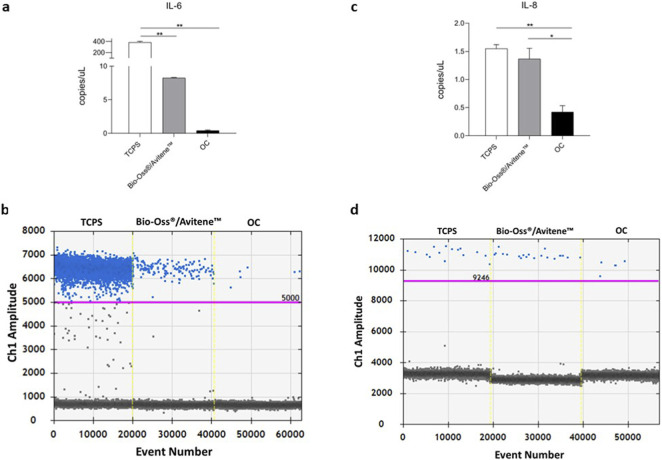
Effect of biomaterial on the gene expression of pro-inflammatory cytokines IL-6, IL-8. **(a,c)** The expression level of pro-inflammatory cytokines IL-6 and IL-8 (mRNA) in BM-MSCs grown on the material (Bio-Oss®/Avitene™), osteogenic medium (OC), and control (TCPS) using ddPCR approch. Gene expression levels are represented as copies/ul (*p < 0.05; **p < 0.001; ANOVA test). **(b,d)** Representative plot of ddPCR amplifications of IL-6 and IL-8. Experiments were performed in technical triplicate for each biological sample (n = 3).

Pro-inflammatory cytokine/chemokine *IL-6* and *IL-8* (mRNAs) were detected in BM-MSCs exposed to osteogenic medium and scaffold, at day 7, compared to TCPS control with ddPCR approach (Figure 2). In BM-MSCs grown on Bio-Oss®/Avitene™ it has been observed a statistical decrease of *IL-6* compared to the control, represented by BM-MSCs grown on the plastic vessel TCPS (**p < 0.001), as well as in in BM-MSCs exposed to osteogenic medium (**p < 0.001) (Figures 2a,b). On the other hand, *IL-8* mRNA levels were significantly decreased in BM-MSCs exposed to osteogenic medium at day 7, compared with both scaffold-cultured BM-MSCs (*p < 0.01) and the TCPS control (**p < 0.001), as shown in Figures 2c,d.

### 3.3 Bio-Oss®/Avitene™ biomaterial modulates the inflammatory protein response in BM-MSCs

**FIGURE 3:**
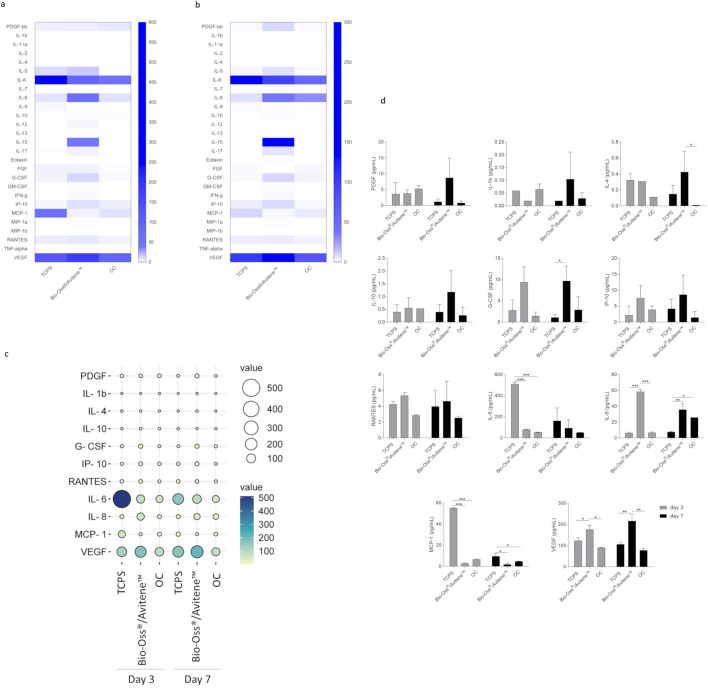
Profile of cytokines/chemokines protein released by BM-MSCs grown on scaffold. Expression levels of cytokines/chemokines released by BM-MSCs grown on biomaterial at days 3 and 7, compared to BM-MSCs grown on plastic vessels (TCPS) and in osteogenic conditions (OC). Supernatants were analysed with Bio-Plex Pro Human Cytokine 27-Plex Immunoassay. Heatmap of cytokines/chemokines released by BM-MSCs at day 3 **(a)**. The colour scale range is 0–600 pg/mL. Heatmap of cytokines/chemokines released by BM-MSCs at day 7 **(b)**. The colour scale range is 0–300 pg/mL. Each row represents a cytokine/chemokine, growth factor whereas each column represents a sample. **(c)** Common cytokines, chemokines, and growth factors released by (i) BM-MSCs grown on (ii) Bio-Oss®/Avitene™ biomaterial, in (iii) osteogenic condition and in basal medium (TCPS, the control) at day 3 and 7 of analysis. The colour and the size of the circles indicated the reliability of the prediction results. The name of each protein was shown on the left. The name of samples was shown at the bottom. **(d)** The common proteins released were represented by black and grey histograms (*p < 0.05; **p < 0.001; ***p < 0.0001; ANOVA test). Experiments were performed in technical triplicate for each biological sample (n = 3).

BM-MSCs grown on Bio-Oss®/Avitene™ composite scaffold, in basal medium and osteogenic condition were analysed, together with supernatants, at day 3 and 7 (Figure 3; Supplementary Table S1, S2). Cytokines/chemokines/growth factors appeared to be dysregulated in BM-MSCs grown on Bio-Oss®/Avitene™ biomaterial and osteogenic medium, compared to the control (TCPS), at day 3 and 7 (Figure 3a,b respectively). Our results showed that some cytokines/chemokines/growth factors (n = 7), i.e., PDGF, IL-1b, IL-4, IL-10, G-CSF, IP-10 and RANTES, although at low concentrations (pg/mL), were expressed by BM-MSCs grown (i) on Bio-Oss®/Avitene™ biomaterial, (ii) in osteogenic condition and (iii) in basal medium (TCPS, the control) at both time-points of analysis (Figure 3c). On the other hand, high expression (pg/mL) of some proteins (n = 4), i.e., IL-6, IL-8, MCP-1 and VEGF, was revealed in BM-MSCs, as reported in Figure 3C. Specifically, Bio-Oss®/Avitene™ scaffold stimulates an increase of cytokines/chemokines/growth factors release, i.e., IL-4, G-CSF, IL-8, VEGF in BM-MSCs (Figure 3d). Indeed, at day 7, a significant increase of cytokine IL-4 was measured in BM-MSCs grown on Bio-Oss®/Avitene™ biomaterial (*p < 0.05). On the other hand, IL-8 was upregulated in BM-MSCs grown on Bio-Oss®/Avitene™ composite scaffold, as well as in the osteogenic condition, compared to control. Interestingly, our data showed that VEGF protein tested upregulated by composite material in BM-MSCs compared to TCPS, as well as under osteogenic conditions at day 3 and 7 (*p<0.05 and **p<0.001, respectively). Moreover, BM-MSCs grown on Bio-Oss®/Avitene™ composite showed a significant increase of G-CSF, compared to control, at day 7 (*p < 0.05).

Monocyte chemoattractant protein-1 (MCP-1) tested downregulated in BM-MSCs grown on Bio-Oss®/Avitene™ biomaterial, compared to control. In our study, the pro-inflammatory cytokine IL-6 was downregulated in stem cells grown on the scaffolds in the early stage (***p < 0.0001) (Figure 3d). In agreement, the level of *IL-6* (mRNA) was lower compared to control, at day 7 (Figures 2a,b). Osteogenic medium modulates the release of cytokines/chemokines/growth factors in BM-MSCs. Indeed, IL-6 tested downregulated, compared to control (TCPS), in osteogenic conditions at day 3 (***p < 0.0001), whereas MCP-1 resulted downregulated at both time-points of analysis, i.e., day 3 (***p < 0.0001) and day 7 (*p < 0.05) (Figure 3d). IL-8 was upregulated in cells grown in osteogenic condition (*p < 0.05) at day 7 (Figure 3d). Finally, among Cytokines/chemokines/growth factors (n = 27) assayed, IL-1ra, IL-2, IL-7, MIP-1b, and TNF-alpha were not expressed by BM-MSCs at day 3. The same cytokines IL-1ra, IL-2, IL-7, MIP-1b, and TNF-alpha, together with IL-13, were not detected in either experimental group, at day 7.

### 3.4 Bio-Oss®/Avitene™ biomaterial promotes bone regrowth in patients

**FIGURE 4:**
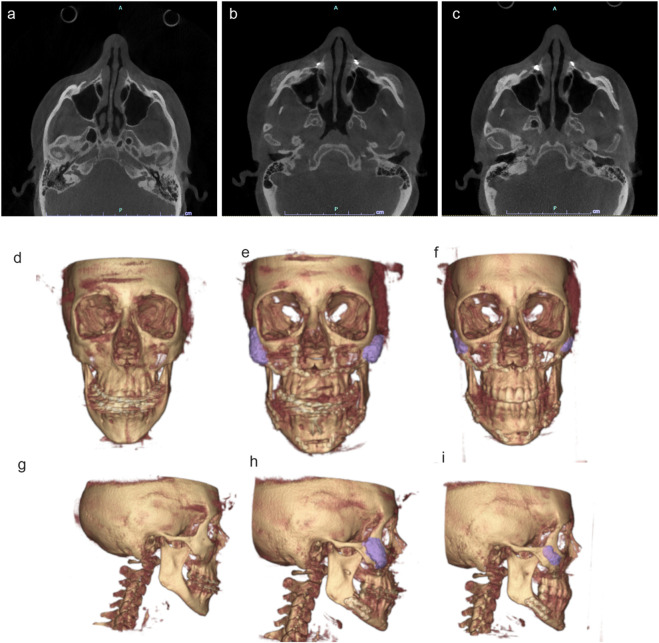
Radiologic analysis of scaffold in patients. a-c, Cone-beam tomogram, coronal slice, at **(a)** T0 (1 week before implant), **(b)** T1 (15 days), and **(c)** T2 (12 months) after surgery. Progressive loss of definition of the granular architecture, with an almost complete radiopacity and apparent corticalization of bone in contact with the prosthesis. The interface between the prosthesis and bone at T2 appears indistinguishable. **(d-i)** Three-dimensional (3D) visualization and the zygomatic implant design. **(d,g)** 3D reconstruction before prosthesis implantation in patients with flat or inadequate projection or asymmetry of the zygomatic area. The 3D visualization shows the placement of prostheses (violet) composed of bony biomaterial (Bio-Oss®) and Microfibrillar Collagen (Avitene™) at day 15 **(e,h)** and 12 months **(f,i)** after implant.

The bone substitute offered remarkable aesthetic results in terms of naturalness and symmetry. Moreover, this kind of prosthesis is not subject to infection nor displacement after osteointegration, as it becomes part of the skeleton itself. The mean dimension of the prostheses calculated on patients’ CBCT (n = 30) was 6.8 mm × 27.9 mm x 16.0 mm with a mean volume of 1,793 mm^3^ (Figure 4). The Bio-Oss®/Avitene™ prosthesis appeared relatively radiolucent compared with the compact portion of the zygomatic bone in the first postoperative CT. It seemed firmly adherent to the underlying zygomatic bone in all patients and the granular structure was still discernible. At the follow-up, the prosthesis had evolved into a radiopacity similar to that of the compact native bone, making the prosthesis–bone interface indistinguishable. At the end of the main orthognathic surgical procedure, utilizing the same upper vestibular incision of the Lefort I through a subperiosteal dissection of the area between infraorbital nerves medially and the zygomatic arch laterally, a pocket was created over the zygomatic bone similar in size to the prosthesis, which was settled without any need of fixation. The three-dimensional (3D) visualization shows the 3D reconstruction and the zygomatic implant design (Figure 4). The patients’ clinical evaluation allows us to postulate important features of the scaffold. CBCT assessment on patients (n = 30), at day 15, showed stable results that could guarantee adequate long-term aesthetic restoration of the zygomatic area. In Supplementary Figure S1, the clinical evaluations of patients before and after surgery are shown for all assessed conditions.

## 4 Discussion

In this study, the immunomodulatory effects of the innovative composite material Bio-Oss®/Avitene™ on BM-MSCs, and bone regrowth in patients, were investigated. Indeed, our investigation aimed to analyse *in vitro* the stem cell immune-response and their secretory profiles induced by composite biomaterial Bio-Oss®/Avitene™, as well as the regenerative potential of the scaffold in a cohort of maxillofacial patients. The *in vitro* model was designed to explore the interaction between the biomaterial and BM-MSCs, mimicking early cellular events following implantation and prospectively exploring the potential use of exogenous cells *in vivo*. BM-MSCs were selected as a well-established and extensively characterized *in vitro* model for studying immunomodulatory and regenerative processes, while acknowledging that dental-derived MSCs represent a promising, tissue-specific alternative that may further enhance the clinical relevance of future investigations. Among the cytokines commonly evaluated *in vitro* to assess the potential immunogenicity of biomaterials, IL-6 and IL-8 are frequently included (Lock et al., 2019). A significant decrease of *IL-6* mRNA was detected in BM-MSCs grown on Bio-Oss®/Avitene™ composite biomaterial and into osteogenic condition, compared to control, at day 7, in agreement with data of our previous study (Mazzoni et al., 2020). At protein level, a significant decrease of released IL-6 was detected in BM-MSCs grown on Bio-Oss®/Avitene™ composite biomaterial, compared to control, at day 3. It has been reported that IL-6 inhibits osteoblastogenesis and matrix mineralization by downregulating osteoblastogenic markers, such as RUNX2, OSX, and OCN in murine calvarial osteoblasts and MC3T3-E1 pre-osteoblastic cells (Kaneshiro et al., 2014) and it is responsible for the defective osteogenesis of osteoporotic BMSCs (Li X. et al., 2016). In this context, the scaffold, used herein, may support bone formation and inhibit immune response at the same time.

Regarding *IL-8,* ddPCR analysis did not show significant dysregulation of the expression of interleukin *IL-8* between BM-MSCs grown on the scaffolds and TCPS. At the protein level, IL-8 was upregulated in BM-MSCs grown on Bio-Oss®/Avitene™ composite scaffold, as well as in the osteogenic condition, compared to control. Although classically considered a pro-inflammatory chemokine, plays a key role in the recruitment of immune and progenitor cells to the site of injury. IL-8 may trigger *in vitro* migration of BM-MSCs, via CXCR2-mediated PI3k/Akt signalling pathway, and enhances osteogenesis in mice (Yang et al., 2018).

Bio-Oss®/Avitene™ composite biomaterial, as well as the osteogenic condition, modulates the expression of other proteins involved in immune response. IL-13, together with IL-4 and IL-10, is a crucial regulator that counterbalances pro-inflammatory signals and facilitates the resolution of inflammation (Al-Qahtani et al., 2024). It has been reported that MSCs can polarize human macrophages into anti-inflammatory phenotype, releasing cytokines, such as IL-4 (Adutler-Lieber et al., 2013), which is typically associated with anti-inflammatory and pro-regenerative responses. Furthermore, exogenous IL-4 has been shown to suppress NLRP3 inflammasome activation by driving the polarization of M1 toward M2 macrophages, thereby facilitating bone repair in aged rat models with bone defects (Li et al., 2023). As reported above, bone has a well-known innate ability to rebuild itself upon damage. However, where the injury is significant, the process of physiological regeneration may be hampered. One of the primary causes of this limit is represented by the incapacity to form a new vascular network that guarantees the flow of nutrients and oxygen, resulting in a necrotic core and non-junction bone. It is well known that the direct effect of VEGF is to trigger angiogenesis and subsequent neovascularization (Dreyer et al., 2021). The indirect effect of the VEGF is the initiation of MSCs into following the osteogenic lineage (Li B. et al., 2016). Interestingly, our data showed that VEGF protein tested upregulated by composite material in BM-MSCs, suggesting that the scaffold may induce neovascularization. Kaigler et al. demonstrated that a single bolus of VEGF delivered without a scaffold did not improve vasculogenesis or bone formation, whereas controlled, slow release of VEGF from hydrogel carriers significantly enhanced both angiogenesis and osteogenesis (Kaigler et al., 2013). In a therapeutic perspective, fine-tuning VEGF concentrations in the local signalling microenvironment is critical for maintaining the physiological balance between accelerated vascular invasion and subsequent bone formation (Grosso et al., 2023). Moreover, BM-MSCs grown on Bio-Oss®/Avitene™ composite showed a significant increase of G-CSF, compared to control, at day 7. G-CSF was shown to stimulate bone regeneration and mobilization of vascular and osteogenic progenitor cells (Roseren et al., 2021). MCP-1 tested downregulated in BM-MSCs grown on Bio-Oss®/Avitene™ biomaterial, compared to control. MCP-1, which owns a potent chemotactic activity for monocytes (Mulholland et al., 2019), is a member of the CC-motif chemokine family, including CCL2, which composes a large group of cellular signalling molecules and cognate receptors. For clinical evaluation, a combination of bony biomaterial (Bio-Oss®) and Microfibrillar Collagen (Avitene™) can be successfully used to mold prostheses for aesthetic cheekbone augmentation during orthognathic surgery in patients with inadequate projection or asymmetry of the zygomatic area. The bone substitute offered remarkable aesthetic results in terms of naturalness and symmetry (D’Agostino et al., 2025). Moreover, this kind of prosthesis is not subject to infection nor displacement after osteointegration, as it becomes part of the skeleton itself. Patients’ clinical evaluation allows us to postulate important features of the scaffold. The CBCT assessment on patients showed stable results that could guarantee adequate long-term aesthetic restoration of the zygomatic area. A qualitative analysis was performed due to the boundaries between the scaffold and the native bone cannot be identified in a precise way due to the osteo-integration phenomenon. Further studies may adopt other strategies to effectively compare the malar volumes before and after malar augmentation. Compared to other scaffolds commercially available, the composite material Bio-Oss®/Avitene™ has the advantage of being customized, as it is created based on of the individual patient morphology/characteristic, thus ensuring a novel personalized medicine approach. In our previous study, more than 400 patients who underwent zygomatic augmentation with Hydroxyapatite/collagen composite prosthesis, together with maxillo-mandibular osteotomy, were evaluated. The clinical, radiological, and histological outcomes observed in that cohort were comparable to those reported in the present study. The initial inflammatory response supports the bone tissue repair and regeneration upon biomaterial implantation. On the other hand, a prolonged or severe inflammation may negatively impact the healing process. It may result in poor tissue remodelling, fibrous scar tissue formation, delayed wound healing, and the destruction of healthy tissue (Batool et al., 2021). In the present work, the clinical application involves an acellular scaffold, and therefore the *in vitro* findings should be interpreted as mechanistic insights rather than a direct translation to the *in vivo* setting.

In summary, our results demonstrate that the Bio-Oss®/Avitene™ scaffold modulates the release of various cytokines and chemokines, notably reducing pro-inflammatory interleukins while enhancing the levels of growth factors associated with stem cell recruitment and angiogenesis. The decrease in pro-inflammatory activity demonstrates that the developed scaffold creates a controlled immune response, supporting healing and regeneration rather than chronic inflammation thereby enhancing its potential for effective tissue reconstruction. The persistence of some factors, such as IL-4 and IL-8, may contribute positively to tissue regeneration by promoting vascularization and orchestrating cellular recruitment and healing processes. In terms of naturalness and symmetry, the bone substitute gave remarkable aesthetic results in patients. Our data indicate that the scaffold has immunomodulatory potential and is capable of directing anti-inflammatory, immune-mediated responses associated with tissue regeneration.

## Data Availability

The raw data supporting the conclusions of this article will be made available by the authors, without undue reservation.

## References

[B1] Adutler-Lieber S. Ben-Mordechai T. Naftali-Shani N. Asher E. Loberman D. Raanani E. (2013). Human macrophage regulation via interaction with cardiac adipose tissue-derived mesenchymal stromal cells. J. Cardiovasc Pharmacol. Ther. 18, 78–86. 10.1177/1074248412453875 22894882

[B2] Al-Qahtani A. A. Alhamlan F. S. Al-Qahtani A. A. (2024). Pro-inflammatory and anti-inflammatory interleukins in infectious diseases: a comprehensive Review. TropicalMed 9, 13. 10.3390/tropicalmed9010013 38251210 PMC10818686

[B3] Arnett G. W. D’Agostino A. Grendene E. McLaughlin R. P. Trevisiol L. (2022a). Combined orthodontic and surgical open bite correction: principles for success. Part 2. Angle Orthod. 92, 431–445. 10.2319/123121-959.1 35293981 PMC9235378

[B4] Arnett G. W. Trevisiol L. Grendene E. McLaughlin R. P. D’Agostino A. (2022b). Combined orthodontic and surgical open bite correction. Angle Orthod. 92, 161–172. 10.2319/101921-779.1 34986216 PMC8887413

[B5] Batool F. Özçelik H. Stutz C. Gegout P.-Y. Benkirane-Jessel N. Petit C. (2021). Modulation of immune-inflammatory responses through surface modifications of biomaterials to promote bone healing and regeneration. J. Tissue Eng. 12, 20417314211041428. 10.1177/20417314211041428 34721831 PMC8554547

[B6] Corduff N. Goldie K. (2025). The Immunologic Spectrum of Biostimulators and its clinical importance. Plastic Reconstr. Surg. - Glob. Open 13, e7001. 10.1097/GOX.0000000000007001 40765684 PMC12323983

[B7] De Pace R. Iaquinta M. R. Benkhalqui A. D’Agostino A. Trevisiol L. Nocini R. (2025). Revolutionizing bone healing: the role of 3D models. Cell Regen. 14, 7. 10.1186/s13619-025-00225-1 40113735 PMC11926310

[B8] Dreyer C. H. Jørgensen N. R. Overgaard S. Qin L. Ding M. (2021). Vascular Endothelial growth factor and mesenchymal stem cells revealed similar bone formation to Allograft in a Sheep model. BioMed Res. Int. 2021, 6676609. 10.1155/2021/6676609 33763484 PMC7946458

[B9] D’Agostino A. Trevisiol L. Favero V. Gunson M. J. Pedica F. Nocini P. F. (2016). Hydroxyapatite/collagen composite is a reliable material for malar augmentation. J. Oral Maxillofac. Surg. 74, 1238.e1–1238.e15. 10.1016/j.joms.2016.01.052 26954559

[B10] D’Agostino A. Trevisiol L. Lobbia G. Galiè M. Battaglini E. Bersani M. (2025). Orthognathic surgery satisfaction following FAB treatment. J. Cranio-Maxillofacial Surg. 53, 608–617. 10.1016/j.jcms.2025.01.023 39919988

[B11] Fabbri E. Brognara E. Montagner G. Ghimenton C. Eccher A. Cantù C. (2015). Regulation of IL-8 gene expression in gliomas by microRNA miR-93. BMC Cancer 15, 661. 10.1186/s12885-015-1659-1 26449498 PMC4598972

[B12] Fedorov A. Beichel R. Kalpathy-Cramer J. Finet J. Fillion-Robin J.-C. Pujol S. (2012). 3D slicer as an image computing platform for the Quantitative imaging network. Magn. Reson. Imaging 30, 1323–1341. 10.1016/j.mri.2012.05.001 22770690 PMC3466397

[B13] Grosso A. Lunger A. Burger M. G. Briquez P. S. Mai F. Hubbell J. A. (2023). VEGF dose controls the coupling of angiogenesis and osteogenesis in engineered bone. Npj Regen. Med. 8, 15. 10.1038/s41536-023-00288-1 36914692 PMC10011536

[B14] Hortensius R. A. Harley B. A. (2016). Naturally derived biomaterials for addressing inflammation in tissue regeneration. Exp. Biol. Med. (Maywood) 241, 1015–1024. 10.1177/1535370216648022 27190254 PMC4950363

[B15] Iaquinta M. R. Torreggiani E. Mazziotta C. Ruffini A. Sprio S. Tampieri A. (2021). *In vitro* Osteoinductivity Assay of hydroxylapatite scaffolds, obtained with Biomorphic Transformation processes, assessed using human adipose stem cell cultures. IJMS 22, 7092. 10.3390/ijms22137092 34209351 PMC8267654

[B16] Iaquinta M. R. Martini F. D’Agostino A. Trevisiol L. Bersani M. Torreggiani E. (2022). Stem cell fate and immunomodulation promote bone regeneration via composite Bio-Oss®/AviteneTM biomaterial. Front. Bioeng. Biotechnol. 10, 873814. 10.3389/fbioe.2022.873814 35832412 PMC9271820

[B17] Kaigler D. Silva E. A. Mooney D. J. (2013). Guided bone regeneration using Injectable vascular Endothelial growth factor Delivery Gel. J. Periodontology 84, 230–238. 10.1902/jop.2012.110684 22668339 PMC3669541

[B18] Kaneshiro S. Ebina K. Shi K. Higuchi C. Hirao M. Okamoto M. (2014). IL-6 negatively regulates osteoblast differentiation through the SHP2/MEK2 and SHP2/Akt2 pathways *in vitro* . J. Bone Min. Metab. 32, 378–392. 10.1007/s00774-013-0514-1 24122251

[B19] Li B. Wang H. Qiu G. Su X. Wu Z. (2016a). Synergistic effects of vascular Endothelial growth factor on bone Morphogenetic proteins induced bone formation *in vivo*: Influencing factors and future research Directions. BioMed Res. Int. 2016, 1–11. 10.1155/2016/2869572 28070506 PMC5187461

[B20] Li X. Zhou Z.-Y. Zhang Y.-Y. Yang H.-L. (2016b). IL-6 contributes to the defective osteogenesis of bone marrow stromal cells from the Vertebral body of the Glucocorticoid-induced osteoporotic Mouse. PLoS One 11, e0154677. 10.1371/journal.pone.0154677 27128729 PMC4851291

[B21] Li Q. Lei X. Wang X. Cai Z. Lyu P. Zhang G. (2019). Hydroxyapatite/collagen three-dimensional Printed scaffolds and their osteogenic effects on human bone marrow-derived mesenchymal stem cells. Tissue Eng. Part A 25, 1261–1271. 10.1089/ten.TEA.2018.0201 30648467

[B22] Li D. Li X. Zhang J. Tang Z. Tian A. (2023). The immunomodulatory effect of IL-4 accelerates bone substitute material-mediated osteogenesis in aged rats via NLRP3 inflammasome inhibition. Front. Immunol. 14, 1121549. 10.3389/fimmu.2023.1121549 37153554 PMC10157059

[B23] Li J. Qu Y. Chu B. Wu T. Pan M. Mo D. (2025). Research Progress on biomaterials with immunomodulatory effects in bone regeneration. Adv. Sci. 12, e01209. 10.1002/advs.202501209 40799152 PMC12412535

[B24] Lock A. Cornish J. Musson D. S. (2019). The role of *in vitro* immune response assessment for biomaterials. JFB 10, 31. 10.3390/jfb10030031 31336893 PMC6787714

[B25] Mazzoni E. D’Agostino A. Manfrini M. Maniero S. Puozzo A. Bassi E. (2017). Human adipose stem cells induced to osteogenic differentiation by an innovative collagen/hydroxylapatite hybrid scaffold. FASEB J. 31, 4555–4565. 10.1096/fj.201601384R 28659417

[B26] Mazzoni E. D’Agostino A. Iaquinta M. R. Bononi I. Trevisiol L. Rotondo J. C. (2020). Hydroxylapatite-collagen hybrid scaffold induces human adipose-derived mesenchymal stem cells to osteogenic differentiation *in vitro* and bone regrowth in patients. Stem Cells Transl. Med. 9, 377–388. 10.1002/sctm.19-0170 31834992 PMC7031637

[B27] Mulholland B. S. Forwood M. R. Morrison N. A. (2019). Monocyte chemoattractant protein-1 (MCP-1/CCL2) Drives activation of bone remodelling and skeletal Metastasis. Curr. Osteoporos. Rep. 17, 538–547. 10.1007/s11914-019-00545-7 31713180 PMC6944672

[B28] Oluwole S. A. Weldu W. D. Jayaraman K. Barnard K. A. Agatemor C. (2024). Design principles for immunomodulatory biomaterials. ACS Appl. Bio Mater. 7, 8059–8075. 10.1021/acsabm.4c00537 38922334

[B29] Roseren F. Pithioux M. Robert S. Balasse L. Guillet B. Lamy E. (2021). Systemic Administration of G-CSF accelerates bone regeneration and modulates mobilization of progenitor cells in a rat model of Distraction osteogenesis. IJMS 22, 3505. 10.3390/ijms22073505 33800710 PMC8037338

[B30] Tang D. Chen M. Huang X. Zhang G. Zeng L. Zhang G. (2023). SRplot: a free online platform for data visualization and graphing. PLoS ONE 18, e0294236. 10.1371/journal.pone.0294236 37943830 PMC10635526

[B31] Torreggiani E. Rossini M. Bononi I. Pietrobon S. Mazzoni E. Iaquinta M. R. (2019). Protocol for the long‐term culture of human primary keratinocytes from the normal colorectal mucosa. J. Cell. Physiology 234, 9895–9905. 10.1002/jcp.28300 30740692

[B32] Trevisiol L. Bersani M. Martinez Garza A. Alvarado E. Arnett G. W. D’Agostino A. (2023). Accuracy of virtual surgical planning in bimaxillary orthognathic surgery with mandible first sequence: a retrospective study. J. Cranio-Maxillofacial Surg. 51, 280–287. 10.1016/j.jcms.2023.05.015 37355372

[B33] Xu W.-C. Dong X. Ding J.-L. Liu J.-C. Xu J.-J. Tang Y.-H. (2019). Nanotubular TiO2 regulates macrophage M2 polarization and increases macrophage secretion of VEGF to accelerate endothelialization via the ERK1/2 and PI3K/AKT pathways. Int. J. Nanomedicine 14, 441–455. 10.2147/IJN.S188439 30666106 PMC6330985

[B34] Yang A. Lu Y. Xing J. Li Z. Yin X. Dou C. (2018). IL-8 enhances therapeutic effects of BMSCs on bone regeneration via CXCR2-mediated PI3k/Akt signaling pathway. Cell Physiol. Biochem. 48, 361–370. 10.1159/000491742 30016780

[B35] Zheng X. Chen L. Tan J. Miao J. Liu X. Yang T. (2022). Effect of micro/nano-sheet array structures on the osteo-immunomodulation of macrophages. Regen. Biomater. 9, rbac075. 10.1093/rb/rbac075 36284748 PMC9580515

