# Supplementary material for: Bio-Oss®/Avitene™ composite scaffold promotes maxillofacial bone regeneration via early osteoimmunomodulation of BM-MSCs: an in vitro and clinical study
Source: Front Bioeng Biotechnol. 2026 Jun 10;14:1795343. doi: 10.3389/fbioe.2026.1795343 (PMC13291549; doi:10.3389/fbioe.2026.1795343)
Supplement: Supplementary file 1 [file Table1.docx]

**Table S1. List of proteins released by BM-MSCs at day 3**

|  | **TCPS** | | **Bio-Oss/Avitene** | | **Osteogenic condition** | |
| --- | --- | --- | --- | --- | --- | --- |
|  | **mean** | **SD** | **mean** | **SD** | **mean** | **SD** |
| **PDGF-bb** | 3,68 | 3,49 | 3,79 | 1,13 | 5,27 | 1,07 |
| **IL-1b** | 0,06 | 0,00 | 0,02 | 0,00 | 0,07 | 0,02 |
| **IL-4** | 0,33 | 0,08 | 0,31 | 0,00 | 0,11 | 0,00 |
| **IL-5** | 8,52 | 2,84 | 11,91 | 3,15 | - | - |
| **IL-6** | 510,90 | 16,75 | 80,02 | 3,05 | 54,24 | 1,15 |
| **IL-8** | 6,23 | 1,04 | 58,40 | 2,58 | 6,64 | 0,95 |
| **IL-9** | 2,08 | 0,64 | - | - | - | - |
| **IL-10** | 0,40 | 0,28 | 0,56 | 0,40 | 0,54 | 0,00 |
| **IL-12** | - | - | 1,94 | 1,37 |  | 0,00 |
| **IL-13** | - | - | 0,21 | 0,15 | 0,04 | 0,03 |
| **IL-15** | - | - | 39,43 | 22,32 | - | - |
| **IL-17** | 0,72 | 0,51 | 3,01 | 1,31 | - | - |
| **Eotaxin** | 0,38 | 0,04 | 0,19 | 0,11 | 0,07 | 0,00 |
| **FGF** | 3,35 | 0,97 | 3,01 | 2,13 | 0,71 | 0,50 |
| **G-CSF** | 2,79 | 2,42 | 9,41 | 3,58 | 1,45 | 0,93 |
| **GM-CSF** | - | - | 0,63 | 0,45 | - | - |
| **IFN-g** | 0,31 | 0,22 | 0,82 | 0,13 | - | - |
| **IP-10** | 2,18 | 2,86 | 7,58 | 3,99 | 3,91 | 1,17 |
| **MCP-1** | 55,09 | 0,41 | 2,90 | 0,54 | 6,68 | 0,39 |
| **MIP-1a** | 0,21 | 0,08 | 0,37 | 0,08 | - | - |
| **RANTES** | 4,26 | 0,33 | 5,36 | 0,37 | 2,86 | 0,13 |
| **VEGF** | 123,67 | 13,51 | 176,47 | 19,42 | 90,36 | 0,55 |
