# Supplementary figures and images for: Bio-Oss®/Avitene™ composite scaffold promotes maxillofacial bone regeneration via early osteoimmunomodulation of BM-MSCs: an in vitro and clinical study
Source: Front Bioeng Biotechnol. 2026 Jun 10;14:1795343. doi: 10.3389/fbioe.2026.1795343 (PMC13291549; doi:10.3389/fbioe.2026.1795343)

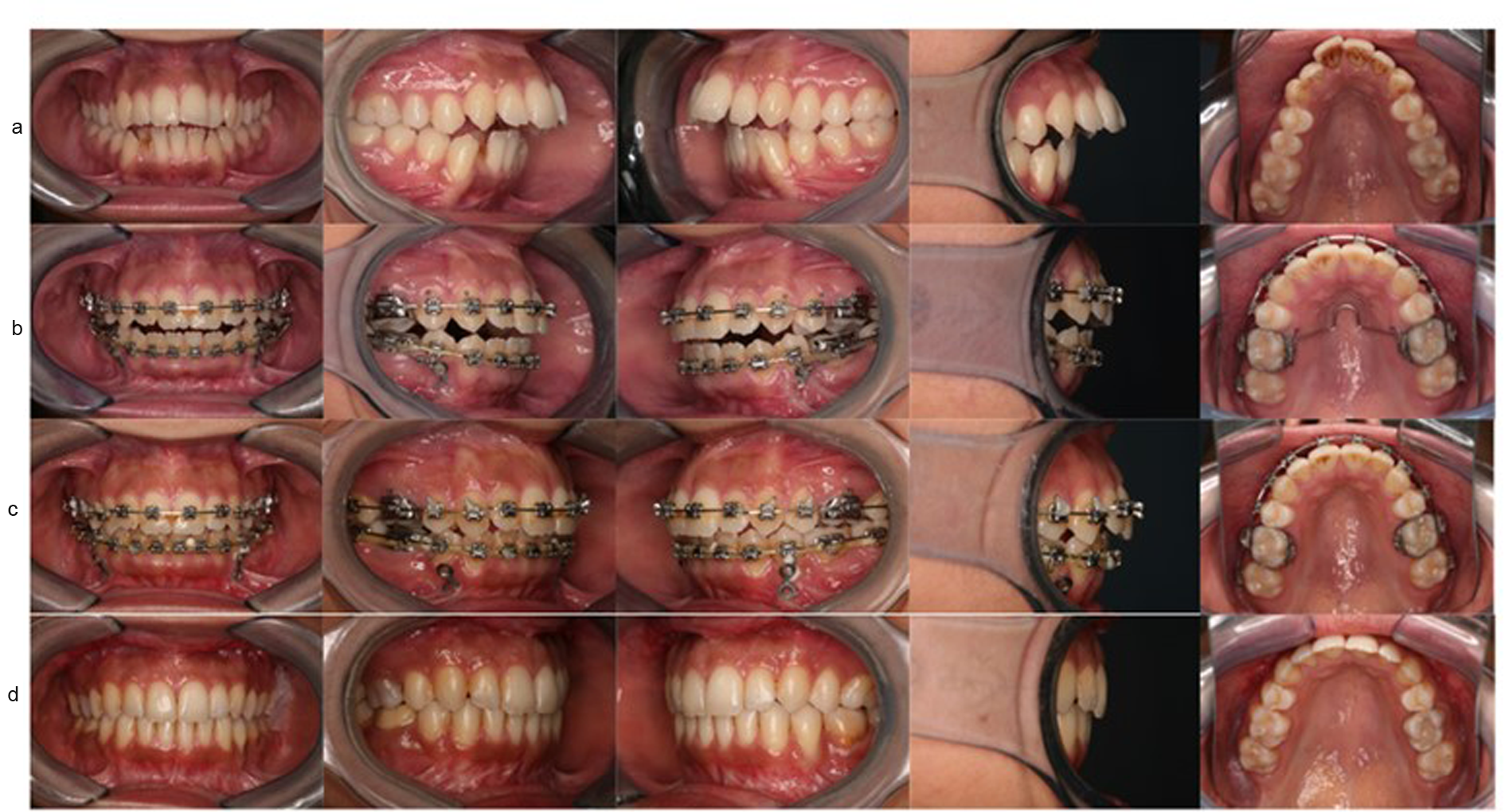

Supplement: Supplementary file 2 [file Image1.tif]
