# Supplementary material for: Bio-Oss®/Avitene™ composite scaffold promotes maxillofacial bone regeneration via early osteoimmunomodulation of BM-MSCs: an in vitro and clinical study
Source: Front Bioeng Biotechnol. 2026 Jun 10;14:1795343. doi: 10.3389/fbioe.2026.1795343 (PMC13291549; doi:10.3389/fbioe.2026.1795343)
Supplement: Supplementary file 3 [file Table2.docx]

**Table S2. List of proteins released by BM-MSCs at day 7**

|  | **TCPS** | | **Bio-Oss/Avitene** | | **Osteogenic condition** | |
| --- | --- | --- | --- | --- | --- | --- |
|  | **mean** | **SD** | **mean** | **SD** | **mean** | **SD** |
| **PDGF-bb** | 1,21 | 0,86 | 8,72 | 6,17 | 0,81 | 0,57 |
| **IL-1b** | 0,02 | 0,00 | 0,11 | 0,11 | 0,03 | 0,02 |
| **IL-4** | 0,15 | 0,11 | 0,43 | 0,26 | 0,01 | 0,00 |
| **IL-5** | 2,17 | 1,53 | 7,04 | 4,98 | 1,45 | 1,03 |
| **IL-6** | 161,80 | 122,05 | 93,71 | 76,22 | 51,72 | 0,26 |
| **IL-8** | 7,56 | 0,84 | 35,72 | 7,66 | 25,85 | 0,00 |
| **IL-9** | 0,99 | 0,70 | - | - | - | - |
| **IL-10** | 0,40 | 0,28 | 1,18 | 0,83 | 0,27 | 0,32 |
| **IL-12** | - | - | - | - | 0,48 | 0,34 |
| **IL-15** | - | - | 143,83 | 101,70 | - | - |
| **IL-17** | - | - | 6,29 | 7,26 | - | - |
| **Eotaxin** | 0,10 | 0,07 | 0,76 | 0,54 | - | - |
| **FGF** | 0,96 | 0,68 | - | - | 1,77 | 1,25 |
| **G-CSF** | 1,08 | 0,76 | 9,68 | 3,50 | 2,89 | 3,07 |
| **GM-CSF** | - | - | 0,65 | 0,46 | - | - |
| **IFN-g** | - | - | 1,55 | 1,10 | - | - |
| **IP-10** | 4,20 | 2,97 | 8,64 | 6,11 | 1,45 | 1,91 |
| **MCP-1** | 9,49 | 2,70 | 1,80 | 1,27 | 4,75 | 0,18 |
| **MIP-1a** | - | - | 0,44 | 0,31 | 0,10 | 0,00 |
| **RANTES** | 3,99 | 2,03 | 4,64 | 2,53 | 2,52 | 0,23 |
| **VEGF** | 106,47 | 7,75 | 216,22 | 32,99 | 77,47 | 8,12 |
